# Analysis of substructural variation in families of enzymatic proteins with applications to protein function prediction

**DOI:** 10.1186/1471-2105-11-242

**Published:** 2010-05-11

**Authors:** Drew H Bryant, Mark Moll, Brian Y Chen, Viacheslav Y Fofanov, Lydia E Kavraki

**Affiliations:** 1 Department of Computer Science, Rice University, Houston, TX, USA; 2 Center for Computational Biology and Bioinformatics, Howard Hughes Medical Institute, Columbia University, New York, NY, USA; 3 Department of Statistics, Rice University, Houston, TX, USA; 4 Department of Bioengineering, Rice University, Houston, TX, USA; 5 Department of Structural and Computational Biology and Molecular Biophysics, Baylor College of Medicine, Houston, TX, USA

## Abstract

**Background:**

Structural variations caused by a wide range of physico-chemical and biological sources directly influence the function of a protein. For enzymatic proteins, the structure and chemistry of the catalytic binding site residues can be loosely defined as a *substructure *of the protein. Comparative analysis of drug-receptor substructures across and within species has been used for lead evaluation. Substructure-level similarity between the binding sites of functionally similar proteins has also been used to identify instances of convergent evolution among proteins. In functionally homologous protein families, shared chemistry and geometry at catalytic sites provide a common, local point of comparison among proteins that may differ significantly at the sequence, fold, or domain topology levels.

**Results:**

This paper describes two key results that can be used separately or in combination for protein function analysis. The Family-wise Analysis of SubStructural Templates (FASST) method uses all-against-all substructure comparison to determine Substructural Clusters (SCs). SCs characterize the binding site substructural variation within a protein family. In this paper we focus on examples of automatically determined SCs that can be linked to phylogenetic distance between family members, segregation by conformation, and organization by homology among convergent protein lineages. The Motif Ensemble Statistical Hypothesis (MESH) framework constructs a representative motif for each protein cluster among the SCs determined by FASST to build *motif ensembles *that are shown through a series of function prediction experiments to improve the function prediction power of existing motifs.

**Conclusions:**

FASST contributes a critical feedback and assessment step to existing binding site substructure identification methods and can be used for the thorough investigation of structure-function relationships. The application of MESH allows for an automated, statistically rigorous procedure for incorporating structural variation data into protein function prediction pipelines. Our work provides an unbiased, automated assessment of the structural variability of identified binding site substructures among protein structure families and a technique for exploring the relation of substructural variation to protein function. As available proteomic data continues to expand, the techniques proposed will be indispensable for the large-scale analysis and interpretation of structural data.

## Background

Understanding the link between protein structure and protein function is a fundamental problem that underlies diverse application areas including drug target identification, protein function prediction, and structure-based evolutionary analysis. The specific few amino acids that mediate the drug-binding affinity of targeted binding sites are an example of a *substructure *within a protein. The catalytic substructures of enzymatic proteins are intrinsically linked to enzyme function [1-4], and establishing a mechanistic understanding of how specific structural features affect protein function is a central problem in structural genomics [5]. The analysis of the physico-chemical properties of the few amino acids constituting these substructures, common to families of functionally related proteins, can provide direct insight to the structural features that dictate a particular enzymatic function [2]. For example, the family of serine proteases is a well-established case of a common functional substructure, the HIS-ASP-SER catalytic triad, dictating a common function in the absence of sequence or fold similarity between chymotrypsins, subtilisins, and lipases [6,7]. Conversely, in the case of TIM barrel proteins which share fold similarity, differing functional substructures within the catalytic site confer differing functions [8]. Therefore, because these functional substructures are essential determinants of protein function, computational approaches to analyze and compare substructures among proteins can provide fundamental insight to the molecular mechanisms that mediate protein function [1,9].

Protein substructures can be represented as *motifs *(*templates*) that abstract the functionally import residues of binding sites. Comparing conserved binding site substructures among all proteins within an enzymatic family can reveal high-level structural trends that may not be apparent if only considering pairs of proteins. The Family-wise Analysis of SubStructural Templates (FASST) method introduced in this work identifies Substructural Clusters (SCs) by comparing the binding site substructures among all proteins within a family. The SCs identified by FASST are demonstrated to reveal substructural patterns that can be associated with phylogeny, conformation change, and homology. Motif Ensemble Statistical Hypothesis testing (MESH), the second method introduced here, exploits the SCs output by FASST to construct multi-structure ensembles of motifs that are shown to have increased function prediction power compared to single-structure motifs. Together, FASST-MESH provides an automated approach for identifying patterns of substructure variation among large numbers of proteins and a method for enriching existing substructure motifs.

Substructure analysis is of practical importance for identifying proteomic drug targets, finding potential drug side-effects, predicting protein function, and evolutionary analysis. Binding site substructures have been considered "receptor-based pharmacophores" [10], allowing a specific few amino acids to indicate likely interaction with a specific ligand-based pharmacophore. Substructural similarity at ligand-binding sites among proteins is indicative of similarity in ligand- and drug-binding properties [3,4]. Exploitation of this property has been applied recently to identify new targets for existing drugs [11] and to computationally analyze potential drug side-effects [10,12]. Specifically, cross-species substructure analysis of binding sites among families of functionally homologous proteins can play an important role in lead evaluation [10,13], and therefore computational approaches to analyze family-wise substructural variation are particularly relevant for modern drug development.

Furthermore, substructure comparison of catalytic sites among proteins has been shown to be a powerful technique for predicting the function of protein structures [7,14,15] and is an important component of structural genomics initiatives that seek to map and functionally annotate the space of protein structures [5,16]. Finally, enzymes evolve under selective pressure to maintain biologically necessary functions [17], and functional substructure conservation in the absence of sequence of fold conservation has been established [18,19]; substructure comparison may be the *only *way to establish homology between proteins that have significantly diverged in both sequence and fold [20]. This work contributes two new computational methods for family-wise substructure analysis that contribute novel approaches to examining protein substructures. Given the biological relevance of substructure analysis and the proliferation of available structures in the Protein Data Bank (PDB) [21], computational approaches to substructure analysis can make meaningful contributions to our understanding of proteomics.

Computational methods for finding functionally significant substructures and methods for comparing substructures to identify biologically relevant proteins with matching substructures are two complementary components of substructure analysis. As far as approaches capable of finding substructures are concerned, earlier work includes ligand-binding cavity identification (CavBase [22], CASTp [23]), structural pattern recognition (GASPS [9], FEATURE [24], FLORA [25]), computational scanning mutagenesis (SNAP [26]), evolutionary analysis (ET [27], ConSurf [28]), expert knowledge (CSA [29]), and automatically curated databases (LigBase [30], SFLD [2], LigASite [31]). Substructures identified by these methods can be computationally represented, either in full or in part, by motifs that model both the geometric and physico-chemical properties of a given substructure. Computationally identifying substructure matches in other proteins with statistically significant similarity to a motif can indicate that a matched protein may share functional characteristics with the motif [7]. Diverse approaches to motif search and/or comparison have been developed and include: SPASM [32], ASSAM [33], PINTS [34], Jess [15], SiteEngine [35], Query3D [36], ProFunc [37,38], ProKnow [39], SitesBase [40], GIRAF [41], MASH [42], LabelHash [43], SOIPPA [20], FEATURE [24], and pevoSOAR [44] to name a few. In general, designing high-quality motifs that accurately capture the functional essence of a substructure is critical and the (successful) performance of motif-driven substructure comparison methods depends directly on the biological relevance of input motifs. The described work complements both the identification and comparison of motifs in novel ways. This paper departs both from finding functionally significant substructures and from comparing substructures to identify biologically relevant matching proteins. The approach presented here combines substructure comparison, unsupervised learning, dimensionality reduction and non-parametric statistical analysis to partition functionally homologous protein families into SCs based upon substructural similarity. This work demonstrates an automated approach that could be used to augment existing substructure motifs already available in repositories such as the Catalytic Site Atlas [29] by geometrically enriching motifs for families that exhibit high structural variability. As both the number and diversity of available structures for a given protein family continue to increase, the enhancement of substructure-based functional annotation methods to accommodate large families is necessary. The automated enrichment of available motifs strengthens the function prediction power of these motifs and facilitates the use of substructure-based analysis methods for large-scale, automated annotation of novel structures.

The biological relevance of the functional substructures modeled by motifs can be exploited for exploratory investigations of the role and structural conservation/variation of a substructure within a large protein family; we demonstrate the utility of this approach using FASST by comparing the SCs output by FASST to biological ontologies such as phylogeny. Furthermore, selecting a single-structure motif as a consensus model of a family-wide functional substructure can prove difficult [1] when functionally conserved protein families become large and species-diverse. The MESH framework transforms single-structure motifs into *motif ensembles *to account for increasing family-wide substructural diversity and provides a robust procedure for identifying statistically significant matches to the motif ensemble as a whole. FASST and MESH directly contribute to substructure-based analysis by providing a motif assessment and refinement procedure. FASST provides an additional avenue of exploratory investigation for selected substructures within a family of interest.

FASST proceeds as follows. For a given enzyme family, a substructure motif of the catalytic site is first defined from a literature reference or other source of substructure motifs [9,22,23,26,29-31,40]. Instances of the motif are then identified in each family member structure by a substructure search algorithm--LabelHash in this paper [43]. Next, all-against-all pairwise Least Root Mean Square Deviation (LRMSD) distance comparisons are computed between family members. The LRMSD of the catalytic site substructure from a given protein to the remainder of the family then encodes the family-wise relationship of the family members to one another as vectors of geometric features. Each geometric feature vector can then be interpreted as a point in a high-dimensional *geometric feature space*, where nearby points in this space indicate similar family-wise relationships for the corresponding substructures. FASST then uses a Gaussian Mixture Model (GMM) clustering approach for unsupervised learning of the SCs. The SCs can then be compared to a biological ontology by mapping meta-data to each substructure for exploratory data analysis.

We demonstrate with FASST that SCs can suggest biological sources of structural variation within a protein family. For the heme-dependent peroxidase family (EC 1.11.1.7) and the xylose isomerases (EC 5.3.1.5), we show that the observed SCs can be explained by the phylogenetic distance between members of the family. Structures of the thermolysin family of bacterial proteases are observed to have catalytic sites with both discrete and continuous modes of flexibility, and structures are known to transition between discrete structural conformation states upon ligation. Analysis of the family-wise structural variety of the serine protease catalytic triad, incorporating over 700 structures from 52 different species and 7 EC classes, demonstrates the ability of FASST to detect substructure variation among convergently related families where the motif substructure resides in many configurations, including some spanning peptide chains. The substructural variation present within each family is automatically identified from the SCs output by FASST.

The FASST method presented here directly complements the *k*-partite [45], bipartite [46,47] and product-graph-max-clique [48] approaches to all-against-all common substructure identification, because these methods can successfully identify common substructures between two [46-48] or more [45] binding sites. The common substructural elements identified by these approaches can serve as a source of new motifs for further substructure analysis. Several of these all-against-all methods have been used to construct "similarity networks" of known ligand binding sites by using pairwise similarity between binding sites in combination with linkage-based [46-48] clustering to build graphs of related sites. However, edges in these "similarity networks" correspond to maximal matches between any given pair of binding sites, causing both the specific subset and number of amino acids compared between a given site and all other sites to *vary *due to differing levels of maximal matches between each binding site pair. Our approach uses a single substructure as a *consistent *point of comparison in every pairwise comparison made within a protein family; hence, the resulting SCs output by FASST can be further utilized, by MESH, to construct a per-cluster representative *consensus motif *that is guaranteed to be found in every cluster member. The substructure-based all-against-all comparison implemented by FASST is most analogous to the seminal work of Holm and Sander [49] on mapping protein fold space via all-against-all Dali comparisons [50]. MESH utilizes the SCs identified by FASST to construct refined substructure motifs that have improved *sensitivity*, and we demonstrate this procedure in a series of protein function prediction experiments. MESH constructs a representative motif for each identified cluster. The collection of representative motifs, for the family, constitutes a single motif ensemble. To provide a statistically rigorous framework for calculating the statistical significance of substructure matches identified by motif ensembles, we introduce a non-parametric model of substructural similarity for multi-structure motifs. When compared to single structure motifs, we demonstrate that the FASST-MESH framework can significantly improve functional annotation sensitivity for structurally diverse families of proteins, while maintaining annotation specificity, for the 15 protein families included in the study.

## Results

The families of proteins included in our study were analyzed with FASST to construct SCs that model the substructural diversity of each family. The underlying source of substructural variation could be clearly attributed to phylogenetic distance, conformation, or protein homology in many cases. The families of proteins we highlight here have a source of substructural variation that can be concretely linked to a single biological factor, in order to better demonstrate the role of each variation source independently. Each structure family was defined by Enzyme Commission (EC) numbers and preference for inclusion into the data set was given to families with a large number of structures. A catalytic site motif was defined for each family from a literature reference (see Table 1) using C_*α *_positions. FASST then takes as input the family and motif and outputs SCs for the family in order to identify the substructural variation within a family. We analyze the SCs of highlighted families in detail below.

**Table 1:** Full protein family dataset used for function prediction experiments.

EC class	PDB ID (Chain)	Amino acid number^Labels^	EC class size
1.1.1.1	1HET (A)	46^*C*^, 48^*S*^, 67^*H*^, 174^*C*^	82
1.1.1.21	1US0 (A)	43^*D*^, 48^*Y*^, 76^*S*^, 77^*K*^, 110^*H*^	89
1.11.1.7	1ARU (A)	52^*RQ*^, 56^*H*^, 57^*D*^, 93^*NR*^, 184^*H*^	83
1.14.13.39	1DWW (A)	194^*C*^, 346^*V*^, 363^*F*^, 366^*W*^, 367^*Y*^	126
2.5.1.18	2A2R (A)	7^*Y*^, 13^*FLR*^, 47^*ACFLM*^, 108^*CFLY*^	190
2.6.1.1	2QA3 (A)	32^*G*^, 34^*G*^, 183^*N*^, 374^*R*^	105
2.7.4.6	1NHK (R)	51^*Y*^, 117^*H*^, 119^*S*^, 128^*K*^	60
3.1.1.7	1H23 (A)	84^*W*^, 117^*G*^, 130^*Y*^, 279^*W*^, 330^*F*^	110
3.1.3.1	1ANI (A)	51^*D*^, 101^*D*^, 102^*S*^, 331^*H*^, 412^*H*^,	44
3.1.3.48	2CM2 (A)	181^*DE*^, 182^*FHMY*^, 216^*S*^, 221^*R*^, 266^*Q*^	248
3.2.1.1	1HT6 (A)	52^*G*^, 178^*R*^, 180^*D*^, 205^*E*^, 291^*D*^	133
3.5.2.6	1YLJ (A)	70^*S*^, 73^*K*^, 130^*S*^, 132^*N*^	254
4.2.1.1	1HCB (A)	94^*H*^, 96^*H*^, 106^*E*^, 119^*H*^, 199^*T*^	282
5.3.1.1	1YPI (A)	12^*K*^, 95^*H*^, 96^*S*^, 165^*A*^	95
5.3.1.5	1DID (A)	53^*H*^, 56^*D*^, 93^*F*^, 136^*W*^, 182^*K*^	71

For each EC class family, a single PDB structure was used to define an input motif. The list of amino acid numbers are documented functional residues found within the primary PDB http://www.pdb.org reference corresponding to each PDB structure. The superscript labels above each amino acid number are the possible amino acid types that can match at each motif point; further details of alternate amino acid label use can be found here [43]. Where multiple amino acid labels per motif point appear, they were determined using ConSurf [28].

### Phylogenetic-based clusters (FASST)

#### Heme-dependent peroxidases

**Figure 1:**
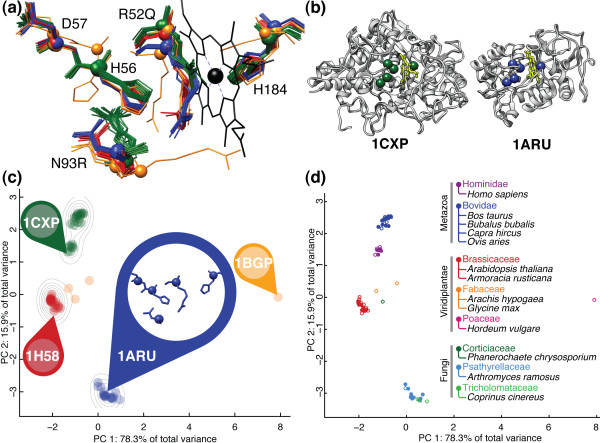
**Substructural Clusters (SCs) for the heme-dependent peroxidases**. **(a) **Superposition of the propagated motifs for the animal and non-animal heme-dependent peroxidases of EC 1.11.1.7 demonstrates geometric diversity. The color of each aligned substructure corresponds to its cluster assignment in (c), and it can be seen that closely aligned substructures in (a) correspond to co-located points in the SCs shown in (c). **(b) **When the backbones of a class II fungal peroxidase [PDB:1ARU] and human myeloperoxidase [PDB:1CXP] are compared, substructural similarity within the heme-binding catalytic site region is evident, but the remainder of the enzyme structures can be seen to have significant topological differences and are assigned to separate topological classes within the CATH structural ontology [54]. **(c) **Applying FASST to the family of peroxidases yields a family-wise geometric feature vector for each catalytic substructure in the family, reducing each substructure shown in (a) to a point in the SCs. Gaussian mixture model (GMM) clustering of geometric feature vectors, projected onto a space of reduced dimension, identifies four clusters denoted by color. The gray isocontours show the smoothed density of substructures in each part of the SCs. **(d) **Substructure positions in the SCs colored by *Family*-level taxanomic classification reveal that phylogenetic distance between proteins is the main source of substructural diversity among the heme-dependent peroxidase binding sites. The open/closed plot characters correspond to apo/holo structures, respectively.

Heme-dependent peroxidases (EC 1.11.1.7) are ubiquitous enzymes responsible for moderating reactions with reactive oxygen species. The lactoperoxidases and myeloperoxidases found in animal leukocytes produce potent antibacterial agents and have been shown to play a role in inflammatory responses [51]. The non-animal class II peroxidases, found in fungi, and class III peroxidases, found in plants, are both secreted enzymes that are thought to play multiple roles including organism development and pathogen defense [52]. The catalytic site region of the *Arthromyces ramosus *class II peroxidase enzyme [PDB:1ARU] includes the proximal (His-184) and distal (His-56) histidines coordinated to the heme group as well as the distal catalytic residues (Arg-52 and Asn-93) and the hydrogen-bonded Asp-57 [53]. Superposition of all of the heme-dependent peroxidase catalytic site structures, identified through motif propagation as outlined in *Methods*, is shown in Figure 1(a). Although the catalytic site motif can be identified within both animal and non-animal peroxidases, geometric variability of the catalytic residues is evident from the alignment. The peroxidase SCs constructed by FASST (see Figure 1(c)) reveal that the peroxidase structures segregate into four main clusters that can be explained well by the phylogenetic ontology of the structures as shown in the corresponding Figure 1(d) plot. The lactoperoxidase structures from *Capra hircus *(goat), *Bos taurus *(cow), *Ovis aries *(sheep), and *Bubalus bubalis *(water buffalo) form a single cluster in the SCs nearby the distinct myeloperoxidase cluster from *Homo sapiens*. The class III plant peroxidases from the *Brassicaceaa Family *form a tight cluster along with the class III plant peroxidases of the *Fabaceae Family *which are near the perimeter, but outside the main cluster. Finally, the class II fungal peroxidases form a fourth distinct cluster most distant from the other three clusters.

The locations of the peroxidase catalytic site substructures in the SCs appear to be highly correlated with the evolutionary history of the enzyme. The animal and non-animal peroxidases are theorized to have originated from two separate endosymbiotic events predating modern plant and animal cells [52]. The sequence identity between the human [PDB:1CXP] and fungal [PDB:1ARU] versions of the enzyme is 9% making a sequence-based approach to analyzing this family as a whole impossible. Pairwise sequence identity between the labeled positions in Figure 1(c) is consistently very low as seen in Table 2. As shown in Figure 1(b), the overall fold topology of the animal and non-animal peroxidases differ greatly and belong to separate fold classes within the CATH structural ontology [54]. However, the catalytic substructure represented by the motif provides a common point of comparison between these peroxidases and allows FASST to identify the significant family-wise catalytic site variation and underlying clusters within the larger protein family. By mapping the SCs to the *Family*-level phylogenetic ontology, FASST is able to propose a hypothetical explanation for the pattern of substructural conservation and variation within the family of peroxidases.

**Table 2:** Pairwise sequence identity between the labeled positions in Figure 1(c) is consistently very low.

	1ARU	1BGP	1H58
1CXP	9%	7%	6%
1ARU	-	14%	7%
1BGP	-	-	40%

#### Xylose isomerases

Metabolic engineering approaches to creating organisms capable of producing biofuels, such as ethanol, from previously unrecoverable plant biomass are being actively studied in the search for renewable energy sources [55]. Xylose isomerase is a key enzyme in many engineered biosynthetic pathways because of its ability to interconvert sugar isomers, allowing novel carbohydrate sources, such as plant biomass, to be utilized over more traditional sugar substrates such as glucose [56]. While members of the peroxidase family demonstrate topological diversity, the family of xylose isomerases (EC 5.3.1.5) are more topologically homogenous, and provide another clear example of SCs that can be linked to the corresponding phylogenetic ontology of the structures.

**Figure 2:**
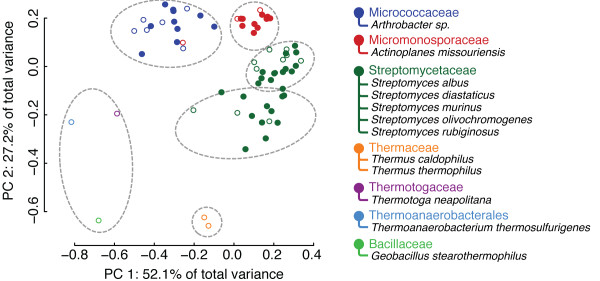
**SCs for the xylose isomerases**. Xylose isomerase structures from 12 different species of bacteria and thermophilic archaea form clusters that can be mapped to the *Family*-level of taxonomic classification. Light gray ellipses denote automatically identified clusters. The open/closed plot characters correspond to apo/holo structures, respectively.

Applying FASST to the catalytic sites of 71 structures of xylose isomerase from 12 different species, including thermophilic archaea and several species of mesophilic bacteria, reveals that variation in catalytic site geometry within the family can be well-explained by the *Family*-level phylogenetic ontology of the family. As shown in Figure 2, the closely-packed, but well-defined clusters of structures clearly map to the phylogenetic labeling at the *Family*-level of taxonomic classification. While the xylose isomerase family exhibits high structural conservation, understanding the substructural relationship between related members of enzymatic families, capable of catalyzing the same reaction under different environmental conditions, is an important step towards rational design of biosynthetic pathways.

### Conformation-based clusters (FASST)

**Figure 3:**
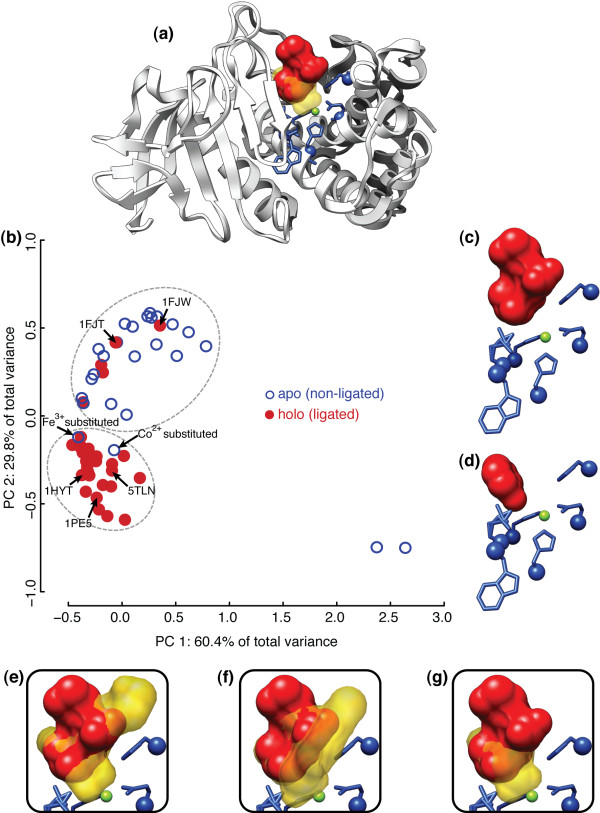
**Ligation-state conformational changes in thermolysin**. **(a) **Backbone of thermolysin structure [PDB:1FJT] with coordinated valine-lysine dipeptide in red and motif residues shown in blue. Side-chains of the motif residues are shown for reference, but only C_*α *_coordinates are used by LabelHash in this paper. The yellow, semi-transparent volume corresponds to the superimposed benzylsuccinic acid ligand of [PDB:1HYT]. The coordinated Zn^2+ ^ion is depicted as a small green sphere in the center of the motif residues. The binding positions of the two ligands are superimposed to illustrate where the occupied regions of the thermolysin binding site differ between the two ligands. **(b) **Applying FASST to the family of thermolysin structures reveals that apo and holo structures segregate into different regions of the SCs. The segregation of structures seen indicates that the motif residues undergo conformational change upon binding a ligand. The location of particular structures in the SCs are labeled for reference. Light gray ellipses denote automatically identified clusters. The open/closed plot characters correspond to apo/holo structures, respectively. **(c), (d) **Holo outlier structures [PDB:1FJT] and [PDB:1FJW] with bound valine-lysine dipeptide and phenol ligands, respectively; the ligand of both structures sits in the side-chain recognition pocket but does not induce conformation change of the motif residues. **(e), (f), (g) **Ligated inhibitors from [PDB:5TLN], [PDB:1PE5], and [PDB:1HYT], respectively, in semi-transparent yellow superimposed with the [PDB:1FJT] binding site. These 3 inhibitors interact directly with the coordinated Zn^2+ ^ion and induce conformational change in the binding site.

Many proteins are known to undergo structural rearrangements and hinge-bending motions upon binding ligands or other proteins. Induced fit via amino acid rearrangements are a common feature of many catalytic sites, and the state of the catalytic site at a given time can often be partitioned into two states: *apo*, an open confirmation with no ligand, and *holo*, a closed confirmation with bound ligand. The thermolysins (EC 3.4.24.27) are a family of bacterial heat-stable metalloproteases that cleave peptide bonds at hydrophobic residue positions and have been shown to change confirmations upon ligand-binding [57]. The family of available thermolysins contains 59 structures of the protein from *Bacillus thermoproteolyticus *and a single structure from both *Staphylococcus aureus *and *Bacillus cereus*, all of which are gram-positive bacteria species (*Bacillales*). Because there are roughly equal numbers of apo (non-ligated) and holo (ligated) structures within the family, and all but two of the structures are repetitions of the same protein from the same species, the effect of ligation state on the substructural variation of the catalytic site can be analyzed in isolation from other possible contributing factors such as phylogenetic distance. Applying FASST to the thermolysins results in the SCs shown in Figure 3(b).

Mapping ligation-state data to the SCs reveals that the clusters determined can largely be explained by the presence/absence of a bound ligand. Outliers revealed by FASST were further investigated to understand why they deviate from the remainder of structures sharing a ligation state. A closer examination of the seemingly misclassified structures reveals that not all ligands binding thermolysin induce conformational change in the binding site substructure (e.g., [PDB:1FJT] and [PDB:1FJW] labeled in Figure 3(b)).

Closer examination of the five holo outlier structures residing within the apo region reveals that either lysine or phenol is bound to the structurally rigid side-chain recognition pocket of these structures in all five cases. In Figure 3(c), the catalytic site of one of the five holo outliers [PDB:1FJT], where a valine-lysine dipeptide is bound near, but not within the catalytic site, is compared to a holo structure with a ligand bound for catalysis in Figure 3(e, f, g). The ligand in Figure 3(e, f, g) can be clearly seen to interact with the catalytic residues as well as the coordinated catalytic metal (Zn^2+^) but the ligand of [PDB:1FJT] is bound just outside of the catalytic site. Binding of the valine-lysine/phenol ligands to the side-chain recognition pocket of thermolysin in the five holo outliers does not induce the catalytic site to alter its geometry, explaining the presence of these holo outliers in the apo region of the plot in Figure 3(b).

Further investigation into the two apo outlier structures, shown to reside in the holo region of Figure 3(b), reveals that these two proteins were artificially modified to coordinate Co^2+ ^and Fe^3+^metals within their catalytic sites, instead of the normal Zn^2+ ^metal found in nature. The substitution of Co^2+ ^and Fe^3+ ^for Zn^2+^alters the geometry of the catalytic site, effectively converting thermolysin into the "closed," ligand-bound holo state [58]. This fact explains why these two artificially substituted apo outliers have higher substructural similarity to the holo structures and are co-located with the holo structures in the SCs shown in Figure 3(b). Therefore, the conformational state of the binding site is a more complete explanation for the SCs determined by FASST, which is highly correlated with, but not completely determined by, the presence/absence of a ligand.

**Figure 4:**
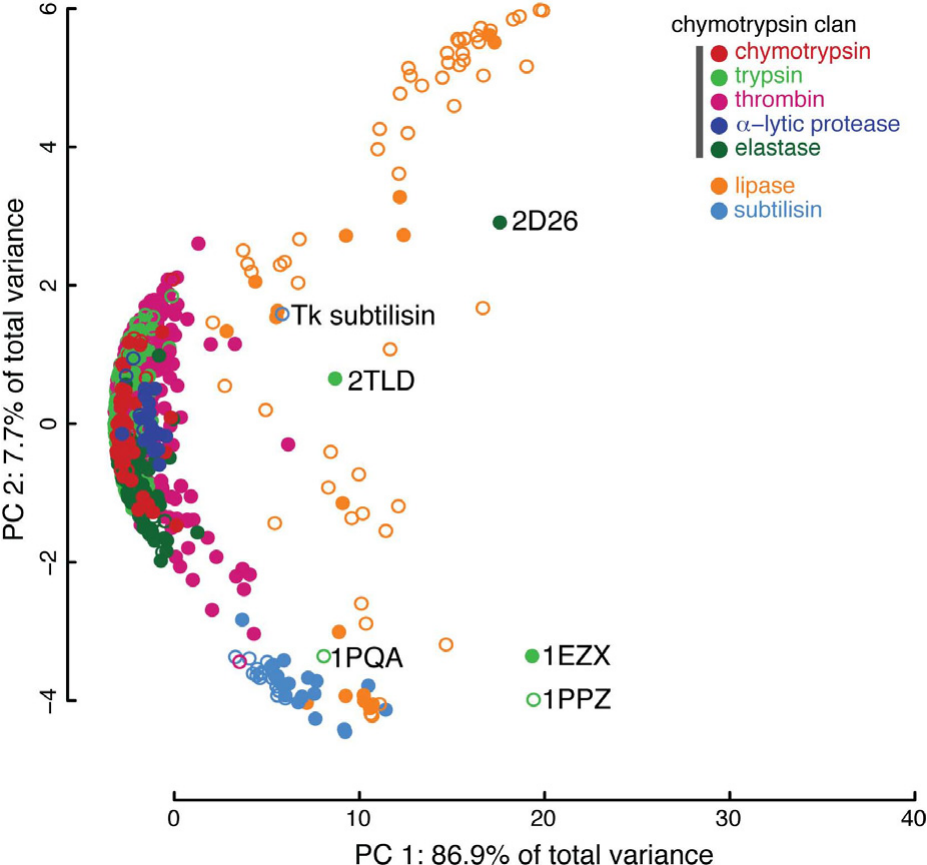
**SCs illustrate catalytic triad diversity among serine proteases**. Comparing the geometry of the ubiquitous HIS-ASP-SER catalytic triad across 730 structures, 52 species, and 7 EC families demonstrates the scalability of FASST to large numbers of structures and the ability of FASST to detect substructure variation among non-divergently related families. All of the divergently-related families of the chymotrypsin clan cluster into a dense sub-group while the convergently-related subtilisin family forms a separate cluster within the SCs. The highly diverse family of lipases form several smaller clusters distinct from both the chymotrypsin-like and subtilisin-like structures. Several trypsin outlier structures are labeled and the references corresponding to each PDB entry document sources of catalytic site deviation. Light gray ellipses denote automatically identified clusters. The open/closed plot characters correspond to apo/holo structures, respectively.

While the presence/absence of a bound ligand is easily determined by examining a protein structure, FASST incorporates only knowledge of the binding site geometry in order to automatically identify each conformation state. As demonstrated by examination of the holo outliers, not all ligands were capable of inducing conformational change in the binding site of thermolysin. The effect of ligation-state within phylogenetic-based clusters was also analyzed for the heme-dependent peroxidases and xylose isomerases to ensure that ligation-state was not influencing the result; open/closed plot characters are used to denote apo/holo structures, respectively, in Figures 1, 2, 3 and 4. When multiple conformations exist within a family of structures, FASST is able to automatically identify the separate conformations as SCs. The conformation-based SCs can then be used as input to MESH to construct a multi-conformation motif ensemble for comparison to non-family structures.

### Homology-based clusters (FASST)

Some protein substructures have proven themselves, throughout the course of evolution, to be so well-suited at catalyzing particular reactions, that they have arisen independently in different kingdoms of life. One such example of convergent evolution in protein substructures is the HIS-ASP-SER catalytic triad which catalyzes the hydrolysis of peptide bonds in many serine proteases [6]. The HIS-ASP-SER catalytic triad is a common substructure among many families of proteases and the geometry of the triad residues across protease families has been shown to be highly conserved [7]. To demonstrate the ability of FASST to detect substructure variation among non-divergently related families where the triad substructure resides in many configurations, including spanning peptide chains, we have considered all of the non-mutant protein structures from the families listed in Table 3 in an analysis of the serine protease catalytic triad. The mutant-filtered family of serine protease structures included 730 protein structures spanning 7 EC classifications and 52 species; the total number of structures in the table is 989 of which 259 are mutant structures. The input motif consisted of the C_*α *_coordinates of the triad residues and was geometrically based upon the [PDB:1ACB] chymotrypsin structure; this motif was able to accurately identify triad residues in all serine protease families, including cases where the triad residues span peptide chains. Correct identification of triad residues for all propagated motifs was subsequently confirmed prior to applying FASST.

**Table 3:** Families of serine proteases, containing the catalytic triad, that were analyzed by FASST.

Family	EC Class	# Structures
Chymotrypsin	3.4.21.1	57
Trypsin	3.4.21.4	355
Thrombin	3.4.21.5	247
*α*-lytic protease	3.4.21.12	39
Elastase	3.4.21.36	90
Triacylglycerol lipase	3.1.1.3	107
Subtilisin	3.4.21.62	94

The chymotrypsin, trypsin, elastase, thrombin, and *α*-lytic protease families are all divergently evolved proteases of the "chymotrypsin clan" (clan SA) [6] and share a common fold that differs from the convergently evolved subtilisin family of proteases. The triacylglycerol lipases have also convergently evolved the serine-based triad and form a third distinct evolutionary group [59]. Application of FASST to the families of serine proteases, as shown in Figure 4, reveals that proteins of the chymotrypsin clan overwhelmingly cluster together with high degrees of overlap in the SCs; the subtilisin structures form a distinct cluster outside of the chymotrypsin clan cluster. Within the chymotrypsin clan, the different families of serine proteases show only subtle variations in triad geometry and are nearly inseparable from one another. It is evident from analysis of the SCs shown in Figure 4 that the lipases exhibit much more catalytic triad geometric variability, overall, than either the subtilisins or chymotrypsins, as they can be seen in many different regions of the space.

Outlier structures within the SCs output by FASST, labeled in Figure 4, were further investigated. One of the most extreme outliers in Figure 4 corresponds to a pancreatic elastase structure [PDB:2D26] complexed with *α*-1 antitrypsin, and this complex was documented to introduce extensive distortion to the catalytic site [60], well-explaining the distant position of this structure from other proteins in the SCs. Similarly, two trypsin outlier structures ([PDB:2TLD] and [PDB:1EZX]) denoted in Figure 4 are complexed with a protein inhibitor that was documented to cause distortion of the catalytic site. Two trypsin structures ([PDB:1PQA] and [PDB:1PPZ]), crystallized with sub-atomic resolution, are also distant from the main chymotrypsin cluster in the SCs [61]. Apo and holo structures are denoted in Figure 4 using open and closed plot characters, respectively, and both apo and holo structures can be found in each cluster identified. The single non-mutant Tk-subtilisin structure, from the archaeon *Pyrococcus kodakaraensis*, is found to be distant from both the chymotrypsin clan cluster and main subtilisin cluster, which suggests a mode of geometric variation different from that of prokaryotic subtilisins and chymotrypsin-like triads. Application of FASST to the serine proteases clearly demonstrates the extremely high degree of both chemical and structural conservation of the catalytic triad across very diverse species and proteins with diverse ligand specificities. Surprisingly, modeling only the triad C_*α *_positions, as was done here, is sufficient to recover the super-family organization of the serine proteases.

### Protein function prediction (FASST-MESH)

FASST provides a method to expose the underlying SCs of a protein family and the MESH framework utilizes the SCs to enhance the function prediction power of substructure motifs. Instead of representing an entire protein family with a single motif, FASST-MESH uses an ensemble of motifs, where each motif within the ensemble is used to represent a cluster within the SCs. MESH automatically constructs a representative consensus motif for each cluster of geometrically related family members output by FASST (see *Methods*). Collectively, the set of consensus motifs for all clusters composes a motif ensemble. Earlier work investigated the performance of averaging all substructures within a family to identify a single family consensus motif [62]. However, it was found that for large geometrically diverse families, a single representative motif, based on any family member substructure or a substructure average of all members, could not sufficiently represent the entire family, just as building a single profile HMM for a large number of distantly related sequences can be difficult. Transitioning to the multiple-model motif ensemble, however, requires that the statistics employed by MESH to distinguish statistically significant matches take into account the presence of multiple tests for significance, one test for each consensus motif in the ensemble (see *Methods*).

**Figure 5:**
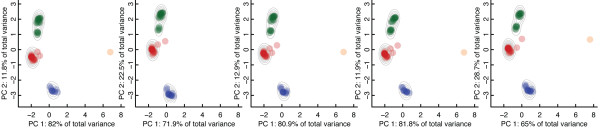
**Cross-fold validation**. Robustness of clusters to data removal during 5-fold cross-validation. During each step of cross-validation, FASST-MESH is used to identify SCs and construct a motif ensemble for the family of peroxidases seen here.

FASST-MESH was used to construct motif ensembles for 15 families of enzymes (see Table 1), as defined by Enzyme Commission (EC) number, and the performance of these motif ensembles was compared to single-structure motifs in a set of function prediction experiments (see Table 4). Function prediction performance can be quantified by *sensitivity*, the percent of True Positives (TP) correctly identified (# TP/(# TP + # FN)), and *specificity*, the percent of True Negatives (TN) correctly identified (# TN/(# TN + # FP)). Because the process of constructing a motif ensemble can be considered *unsupervised learning *of the family substructure space, 5-fold cross-validation was implemented, where the motif ensemble was built from 4/5 of the data and then the last 1/5 was used for performance assessment. The robustness of the SCs identified during cross-fold validation (as shown in Figure 5) can be seen by the stability of the clusters during each of the 5 cross-fold validation steps. Two EC families included in the function prediction experiments are discussed below, and each demonstrates a different extreme of sensitivity/specificity improvement after applying FASST-MESH.

**Table 4:** Function prediction performance of motif ensembles versus single-structure motifs at significance threshold of α = 0.01.

	Single structure motif		Motif ensemble (CV)		**Improvement (x-fold)**	
EC class						
	%Sens. (#TP)	%Spec. (#FP)	%Sens. (#TP)	%Spec. (#FP)	Sens.	Spec.
1.1.1.1	52.4% (43)	99.2% (83)	74.3 ± 7.0% (61)	99.4 ± 0.0% (62 ± 4)	1.4	1.0
1.1.1.21	93.3% (83)	99.1% (146)	93.2 ± 4.8% (83)	99.2 ± 0.1% (136 ± 5)	1.0	1.0
1.11.1.7	91.6% (76)	99.1% (131)	92.7 ± 10.0% (77)	99.5 ± 0.0% (78 ± 8)	1.0	1.0
1.14.13.39	90.5% (114)	99.3% (87)	96.1 ± 2.7% (121)	99.4 ± 0.0% (73 ± 7)	1.1	1.0
2.5.1.18	25.3% (48)	99.1% (171)	46.3 ± 5.1% (88)	99.2 ± 0.0% (140 ± 5)	1.8	1.0
2.6.1.1	66.7% (70)	99.1% (153)	82.9 ± 5.4% (87)	99.3 ± 0.0% (121 ± 5)	1.2	1.0
2.7.4.6	81.7% (49)	99.2% (137)	88.3 ± 2.6% (52)	99.4 ± 0.1% (113 ± 5)	1.1	1.0
3.1.1.7	98.2% (108)	99.2% (82)	99.0 ± 2.0% (108)	99.4 ± 0.0% (60 ± 2)	1.0	1.0
3.1.3.1	84.1% (37)	99.1% (122)	100.0 ± 0.0% (44)	99.3 ± 0.0% (97 ± 6)	1.2	1.0
3.1.3.48	28.6% (71)	99.1% (155)	56.1 ± 3.6% (139)	99.4 ± 0.1% (109 ± 11)	2.0	1.0
3.2.1.1	83.5% (111)	99.1% (149)	88.7 ± 7.9% (117)	99.4 ± 0.1% (102 ± 17)	1.1	1.0
3.5.2.6	35.0% (89)	99.2% (144)	81.2 ± 6.3% (208)	99.4 ± 0.0% (107 ± 9)	2.3	1.0
4.2.1.1	87.9% (248)	99.1% (112)	95.3 ± 3.5% (269)	99.6 ± 0.0% (49 ± 4)	1.1	1.0
5.3.1.1	78.9% (75)	99.1% (143)	82.1 ± 10.9% (78)	99.4 ± 0.1% (100 ± 11)	1.0	1.0
5.3.1.5	97.3% (71)	99.1% (118)	98.5 ± 2.3% (71)	99.4 ± 0.1% (92 ± 11)	1.0	1.0

For each single-structure motif, a motif ensemble was constructed using FASST-MESH. Next to each % sensitivity value is the total number of true positive (TP) matches; next to each % specificity value is the total number of false positive (FP) matches. The performance of motif ensembles was assessed using 5-fold cross-validation and the sensitivity/specificity values correspond to mean ± standard deviation across the 5 folds. The x-fold improvement is calculated as: mean motif ensemble performance divided by single-structure performance.

**Figure 6:**
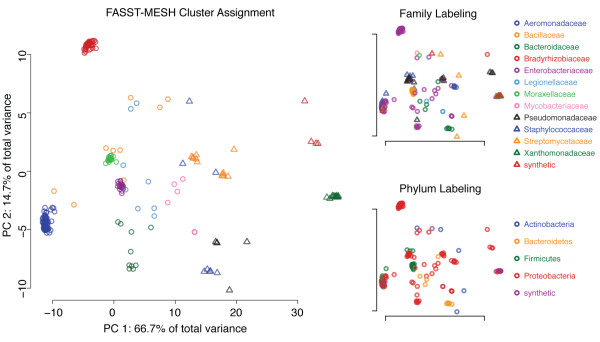
**SCs identified by FASST within the *β*-lactamases**. Applying FASST to expose the substructural diversity of a catalytic substructure among the *β*-lactamases reveals many distinct clusters within the family. The GMM clustering step of FASST identifies 13 sub-groups within the family and the colors/shapes of points in the SCs correspond to cluster assignment. MESH then constructs one consensus motif for each cluster identified, resulting in an ensemble of 13 motifs. Function prediction sensitivity improves from 35.0% (single-structure motif) to 81.2% when using the motif ensemble constructed by FASST-MESH. For the highly diverse family of *β*-lactamases, the SCs output by FASST shows that many distinct sub-groups exist within the family. MESH takes advantage of this information to more completely model the geometric diversity present, thereby improving functional annotation coverage of the family. Mapping *Family*- and *Phylum*-level phylogenetic data to each of the substructures as shown in the corresponding plots on the right reveals that some, but not all, of the clusters identified are due to evolutionary distance between proteins. For example, the *Bacillaceae *proteins can be seen to form a single sub-group while *Enterobacteriaceae *proteins are distributed throughout the SCs in several clusters, indicating that another biological factor is working in concert with phylogenetic distance among the family of *β*-lactamases to produce the structural diversity uncovered by FASST.

The diverse family of *β*-lactamases (EC 3.5.2.6) includes structures from 26 different bacterial species. Using the 13 clusters identified from the SCs output by FASST as shown in Figure 6, MESH constructs a consensus motif for each cluster, resulting in an ensemble of 13 consensus motifs. The *β*-lactamase motif ensemble, constructed by FASST-MESH, identified 81.2% of functionally homologous proteins (as defined by the EC class) with statistically significant substructure matches. The corresponding single-structure *β*-lactamase motif only identified 35.0% of functional homologs, and therefore FASST-MESH improved the functional annotation sensitivity of the single-structure motif by 2.3-fold while maintaining the high specificity of the single-structure motif.

In the family of peroxidases (EC 1.11.1.7) analyzed in Figure 1, a single-structure motif was capable of identifying a statistically significant match for 91.6% of the EC family, and therefore already showed high sensitivity. After applying FASST-MESH to the single-structure peroxidase motif, annotation sensitivity improved only slightly (~1% improvement) but the absolute number of false positive matches identified decreased from 131 to 78 ± 8. The decrease in false positive matches, resulting from use of a motif ensemble, occurred because true positive matches tended to match multiple consensus motifs within the ensemble with low LRMSD, while many false positive matches have only marginally significant LRMSD to a single consensus motif, and applying multiple testing correction to the final set of matches for a given false positive often caused a single marginally significant match to move outside of the significance threshold.

As both the number and diversity of available structures for a given protein family continue to increase, the enhancement of substructure-based function prediction methods to accommodate large families is necessary. This work demonstrates an automated approach (outlined in *Methods*) that could be used to augment existing substructure motifs already available in repositories such as the Catalytic Site Atlas (CSA) [29] by geometrically enriching motifs for families that exhibit high structural variability. The automated enrichment of available motifs by FASST-MESH strengthens the function prediction power of these motifs and facilitates the use of substructure-based analysis methods for large-scale, automated annotation of novel structures.

### Comparison with sequence and whole structure approaches

Similarity among proteins belonging to an enzymatic family can be difficult to detect using sequence and whole structure approaches when such families are sequentially and topologically diverse. The heme-dependent peroxidase and xylose isomerase families differ greatly in the amount of family-wide fold and sequence similarity. To assess the ability of sequence and whole structure (fold) analysis to identify the structures in each family as interrelated, each family was combined with a set of 50 functionally unrelated structures randomly selected from the nrPDB_95_. Additionally, each family was combined with all structures sharing the same SCOP [63] superfamily classification in a separate experiment from the random nrPDB_95 _structures. The heme-dependent peroxidases were combined with all structures within the heme-dependent peroxidase superfamily (SCOP:48113) which includes structures from EC:1.11.1.5 (cytochrome-c peroxidases), EC:1.11.1.6 (catalases), EC:1.11.1.7 (heme-dependent peroxidases), and EC:1.11.1.11 (L-ascorbate peroxidases). The xylose isomerases were combined with all structures from the xylose isomerase-like superfamily (SCOP:51658) which includes structures from EC:5.3.1.5 (xylose isomerases) and EC:5.3.1.14 (L-rhamnose isomerases). Comparing the inter-cluster distance of clusters belonging to a family relative to the distances to functionally unrelated structures illustrates the amount of intra-family similarity that is evident when using each approach.

The sequence and structure comparisons were implemented by using CLUSTALW [64] and Combinatorial Extension (CE) [65], respectively, to compute the pairwise distances between proteins instead of LabelHash (see *Methods (Step 2)*); all remaining steps of FASST were carried out identically for each approach (*Methods (Steps 3-4)*). The non-substructure methods will be referred to as FASST_CLUSTALW _and FASST_CE _hereafter, while FASST will refer only to the substructure-based approach.

The results of FASST applied to the heme-dependent peroxidase and xylose isomerase families, each in combination with the functionally unrelated structures, are shown in Additional files 1 and 2, respectively. In both cases, the substructure-level analysis implemented by FASST identifies the within-family structures to be highly similar to one another (high intra-family similarity) relative to the functionally unrelated structures. These results demonstrate that functionally unrelated structures can be clearly identified as outliers from the remainder of structures in a family analyzed by FASST.

Applying FASST_CLUSTALW _and FASST_CE _to the heme-dependent peroxidases (see Additional file 3) results in multiple clusters of peroxidases and a single, more scattered cluster consisting of unrelated structures. In contrast to the FASST result (Additional file 1), the individual peroxidase clusters identified by FASST_CLUSTALW _and FASST_CE _are as distant from one another as to the functionally unrelated cluster. Using FASST_CLUSTALW _and FASST_CE _to analyze the xylose isomerases (see Additional file 4) results in the within-family structures grouping into multiple clusters well-separated from the functionally unrelated structures; the thermophile xylose isomerase structures are roughly equidistant to the functionally unrelated structures and the remainder of the family.

The average running times of FASST were 4.5 min (FASST), 3.2 min (FASST_CLUSTALW_), and 185.6 min (FASST_CE_); times reported are the wall-clock times for running with a single core on the following system: 2.4 GHz Intel Core 2 Duo, 4GB DDR3 memory, MacBook Pro.

The comparison of FASST with FASST_CLUSTALW _and FASST_CE _demonstrates that intra-family similarity may be more difficult to detect by sequence and fold comparison in some cases. The substructure-level analysis used by FASST can further distinguish functionally related and unrelated structures when conserved substructures can be identified. Therefore, FASST provides a complementary approach that can be used in combination with sequence and fold analysis for analyzing the diversity of functionally related enzymes.

## Discussion

Understanding the significant geometric variability among enzyme catalytic sites is an important component of structural analysis. As the number of solved protein structures grows, methods capable of summarizing and analyzing large amounts of structural data will become increasingly necessary. While whole structure alignment and protein fold analysis can be a valuable tool for assessing protein homology, in the absence of sequence similarity, extremely distantly related enzymes or enzymes which are examples of convergent evolution may be ill-suited to whole structure comparison techniques. However, when no detectable domain or fold homology exists, enzymes are still capable of exhibiting functional equivalence through chemically and geometrically synonymous functional substructures. Techniques capable of assessing the family-wise similarity of these conserved substructures can reveal new insights into the relationships among families of structures. FASST has the ability to recognize modes of family-wise geometric variation among substructures and knowledge of the substructural diversity of a family can guide hypotheses about the role of the substructure in different proteins.

### Biological significance of SCs

In several families of proteins, we have identified possible sources of geometric variation and linked these sources of variation to the substructural clusters automatically identified by FASST. In the peroxidase family, the geometric distance between catalytic sites appears to be correlated with phylogenetic distance. Organisms that are more closely related, such as the plant and fungal species, were shown to have more geometrically similar catalytic sites to one another than to more distantly related phyla, such as vertebrates. With the family of thermolysin structures, we demonstrated how FASST automatically captures modes of catalytic site flexibility, correctly segregating structures into clusters based upon ligation state. Using the families of serine proteases, we demonstrated how FASST extends naturally to very large numbers of structures and is still capable of identifying the major modes of geometric variation across vast numbers of species and triad configurations that include chain spanning and non-spanning instances. Finally, FASST is able to identify structural outliers within families, and these outliers were shown to have biochemical causes for substructural deviation from the remainder of the family, thereby guiding further inquiry to these anomalous structures.

FASST partitions a protein family into self-similar clusters of structures and in doing so, constructs SCs that can then be linked with biological metadata to possibly explain the family-wise diversity. Here we have highlighted particular protein families whose substructural diversity can be clearly linked to a single biological ontology, such as phylogeny, conformation, or homology. In several families included in the function prediction experiments, the sub-groups identified by FASST cannot be clearly attributed to a single biological factor. The *β*-lactamases are an example where some clusters clearly correspond to a single phylogenetic branch of bacteria, but other species of bacteria form multiple, distinct clusters as shown in Figure 6. In the typical case, there are likely multiple biological factors working in concert to produce substructural variability. It is intriguing to combine large-scale metadata analysis with FASST to automatically correlate likely biological factors, such as phylogeny, ligation state, and crystallization conditions, with FASST-identified clusters to unravel more complex relationships among functional substructures.

### Diffierentiating sequential and structural redundancy

Using FASST to analyze a catalytic site substructure of thermolysin among 61 sequence-similar proteins demonstrates how latent biological trends can be identified even within a sequentially-homogenous collection of structures. The thermolysin family examined here contained 59 different structures of the exact same enzyme from *B. thermoproteolyticus *and yet FASST was able to automatically uncover a structural trend where the catalytic substructure modified its position only upon binding ligands that interact directly with the coordinated zinc ion. If only sequentially non-redundant structures were used by FASST, this trend could not have been identified because of the miniscule number of sequentially-distinct crystallographic structures for thermolysin. This result demonstrates the additional information that can be garnered by researchers when all available structures are incorporated into a structural analysis. Similarly, the Multiple Solvent Crystal Structures (MSCS) technique utilizes repeated crystallizations of the same enzyme under different solvent conditions in order to probe for functional sites [66,67]. Several of the available thermolysin structures incorporated in our study were produced as part of MSCS experiments [68,69]. Our work demonstrates that FASST can detect subtle trends among sequentially-similar structure collections and is an important tool for analyzing and understanding structure-function relationships across large numbers of protein structures.

### FASST-MESH improves single-structure motifs

After identifying both the existence and membership of structurally defined clusters within a protein family via the automated FASST-MESH framework, this substructural information can be used to enhance existing substructural motifs in order to more accurately represent large families with diverse catalytic site geometry. Our function prediction experiments show that by representing a structurally diverse family with a motif ensemble, we can better capture the variety of substructures present within a given family and increase function prediction sensitivity while maintaining specificity. In cases where family-wide geometric diversity was found to be low, single structure motifs alone can have high sensitivity. However, even when geometric variability is low, motif ensembles created by FASST-MESH always maintain the function prediction performance of single structure motifs and demonstrate vast improvement in several cases among the families included in our study (see Tables 1 and 4). While LabelHash was used here as the underlying substructure comparison tool, we are not attempting to compare the performance of LabelHash to other comparison tools. Rather, the purpose of the function prediction experiments presented here is to illustrate cases where a single-structure motif insufficiently models a large class of functionally homologous, but structurally diverse proteins, and to demonstrate a method to improve the function prediction sensitivity of motifs in general by using motif ensembles.

### Automated motif definition

In this paper, the substructure motifs given as input to FASST (see Table 1) were constructed only from residues that have been experimentally confirmed to play a role in enzyme function in order to separate the subproblem of motif definition from motif analysis. While the input single-structure motifs used here were manually defined, a multitude of automated approaches to motif definition are possible. Our previous work successfully used evolutionarily conserved residues, as determined by Evolutionary Trace [27], for automated motif definition [42].

Because motifs are an input parameter to FASST, different methods of identifying the residues constituting functional substructures can be used in conjunction with FASST, and by doing so, FASST provides an automated approach to further analyze and understand the role of these substructures. In future work, several substructure selection methods and databases, such as CASTp, ET [27], ConSurf [28], CSA [29], SNAP [26], and LigBase [30], will be used as sources for large numbers of motifs. This work used only residues deemed to be functionally important by experimentalists, as defined by literature references, in order to isolate the performance of FASST-MESH from methods that automate substructure selection.

## Conclusions

FASST has been shown to be a powerful technique for assessing family-wise structural variability among analogous protein substructures. We have demonstrated examples of substructural clusters that can be linked to phylogenetic distance, ligation state, and protein homology. The complementary MESH framework provides a systematic approach to create concise motif ensembles that represent the structural variability within a protein family. Such ensembles can be used to improve function prediction for families with significant structural variability.

Many proteins are known to have structurally conserved, but non-catalytic substructures, such as steric recognition sites, metal/ligand sequestering sites, phosphorylation sites, cofactor binding sites, or immunologically important substructural epitopes. Using the FASST-MESH approach for these non-catalytic substructures can be done without modification to the method because FASST-MESH makes no assumptions about the types of substructures modeled by motifs nor underlying sources of structural variation. Our future, application-specific work will focus on understanding particular structure-function relationships among both catalytic and non-catalytic substructures. As the available number of protein structures continues to rapidly grow, methods for automated, large-scale analysis of structures such as FASST-MESH will be critical for identifying high-level structural trends among proteins and placing newly solved structures in the larger context of existing structural data.

## Methods

The *family-wise *substructure analysis method developed here (FASST) takes as input a user-defined substructure motif and a *family *of protein structures, as defined by EC classification here, and outputs Substructural Clusters (SCs) that identify sub-groups of proteins within the larger family. Subsequent application of MESH to the sub-groups identified by FASST constructs a set of *consensus motifs*, collectively referred to as a *motif ensemble*, that can be used to represent the structural variety of the family for function prediction experiments. The combined FASST-MESH procedure is as follows: **(FASST: Step 1) **using LabelHash [43] (available online at http://labelhash.kavrakilab.org), or another substructure search method (FASST is not tied to a particular search method), compute matches of the user-defined motif to identify analogous substructures in all family members, thereby creating one *propagated motif *per member; **(FASST: Step 2) **compute an all-against-all LRMSD alignment of each propagated motif, yielding a vector of substructure distances for each family member which we call a *geometric feature vector*; **(FASST: Step 3) **perform dimensionality reduction on the set of geometric feature vectors via principal components analysis (PCA) [70] and project each geometric feature vector onto the number of PCs necessary to preserve 90% of the original variance; **(FASST: Step 4) **cluster the dimensionality-reduced geometric feature vectors using a Gaussian Mixture Model (GMM) [71] to create the Substructural Clusters that identify sub-groups within the family; **(MESH: Step 5) **build a set of consensus motifs to represent the clusters of the family by selecting an exemplar structure from each cluster or averaging substructures within a group; **(MESH: Step 6) **for function prediction, match the consensus motifs against a background reference set of unrelated structures (e.g., nrPDB) to search for proteins with substructural similarity to the original structure family. Then, identify statistically significant matches using a non-parametric hypothesis testing framework for substructural similarity [42,72], which is adapted and extended here to accommodate motif ensembles. Each of the steps is outlined in detail below.

### Step 1: motif definition and propagation

To quantify the geometric similarity between a pair of catalytic substructures, the LRMSD distance metric is commonly used, but to model the geometric similarity between a given catalytic site and a family of catalytic site substructures we introduce a simple extension to pairwise LRMSD that will be referred to as geometric feature vectors.

The procedure for building geometric feature vectors begins with a single, user-defined motif, S*, that represents the geometry and chemistry of a shared substructural element within the family. The S* for each of the families included in this study were constructed from documented residues in the literature reference associated with each PDB structure listed in Table 1. For example, S* for the heme-dependent peroxidases includes the C_*α *_atom from each of the following residue numbers with the alternate amino acid labels shown in superscript: 52^*RQ*^, 56^*H*^, 57^*D*^, 93^*NR*^, 184^*H*^; the 3-dimensional coordinates of each C_*α *_∈ S* were taken from [PDB:1ARU] as noted in Table 1 and the residue numbers listed are according to [PDB:1ARU]. Care should be taken to define S* with appropriate amino acid alternate labels; the set of amino acid alternate labels for each motif residue defines the allowed mutations per motif residue used when identifying possible matching substructures. ConSurf [28], was used in this work to identify alternate amino acid labels per motif residue for several motifs in Table 1; the alternate amino acid labels are identified from the per-residue conservation and mutation data output by ConSurf. However, when available, an expert-curated multiple sequence alignment allows for the highest confidence in amino acid alternate selection.

First, the user-defined motif, S*, is matched against a family of *n *protein structures, *F *= {*f*_1_, ..., *f*_*n*_}, as defined by Gene Ontology (GO) terms or Enzyme Classification (EC) levels, for example, to yield a set of matches . In this work, LabelHash [43], was used to identify substructure matches by searching each protein in *F *for similar substructures to the motif, S*. Every match,  is a bijection between S* and a substructure of *f*_*i*_, and defines a unique substructural element within *f*_*i *_that will be referred to as a propagated motif, . A caveat of the propagation step is that there are limits on LRMSD at which a pair of motifs can be confidently recognized as functionally related. The LRMSD threshold for confident propagation can differ significantly depending on both the size and number of alternate amino acid labels (allowed substitutions) contained within the motif. For a detailed analysis of the variance of LRMSD thresholds for different motifs, see [42]. For complete algorithmic details of how LabelHash identifies substructure matches to motifs see [43].

### Step 2: encoding geometric features

The pairwise LRMSD between two propagated motifs will be denoted by  and the geometric feature vector, **g**_*i*_, for a given *f*_*i *_is defined as a vector of LRMSD values: . The set of geometric feature vectors representing all structures in the family, *F*, will be denoted as **G **= {**g**_1_, ..., **g**_*n*_}, and **G **constitutes an all-against-all alignment of the substructures that correspond to each respective protein in *F*. Each **g**_*i *_∈ **G **defines a point in geometric feature space that represents the corresponding *f*_*i *_∈ *F *and it is important to note that structures with similar family-wise distances will be nearby in the geometric feature space. By constructing the geometric feature space of a family, the structural variation present within an all-against-all substructure alignment (as shown in Figure 1(a)) is preserved, but distilled into a much simpler representation that is more amenable to common machine learning techniques such as clustering.

### Step 3: dimensionality reduction

Understanding the family-wise structural information encoded by **G **will lead to the motivation for the following step-dimensionality reduction. Let, for example, *n *= 100 and consider that the geometric feature vectors, **g**_*i *_∈ **G**, will be 100-dimensional, making analysis of the feature space difficult. It is often the case that many structures in a homologous family, as defined by EC or GO for example, will contain several crystallizations of the same protein, from the same species, causing some of the propagated motifs to be nearly identical in geometry. Because of these similar structures, a given **g**_*i *_will have some very highly correlated features that increase the dimensionality of the feature vector representation, but do not each provide orthogonal information about the family-wise relationship of *f*_*i *_to *F *. Removing similar structures via sequence-identity thresholds requires that a representative structure from the sequence-similar set to be selected. However, sequence-identity removal techniques do not consider the geometric diversity of available structures when selecting a representative structure. The method presented here allows all available structures for a family to be included without filtering for sequence identity specifically because of the dimensionality reduction step. By including all available structures in the analysis, the method presented here does not make *a priori *assumptions about the sequential or structural diversity of a family of proteins. While reducing the dimensionality of **G**, it is important to preserve the distances between substructures in feature space, since the purpose of geometric feature encoding is to find sub-groups of related substructures within *F*. We begin by finding the Principle Components (PCs) of **G **and then project **G **into a subspace of the PCs that captures at least 90% of the original variance in **G**; we denote the lower-dimensional projection of **G **as **G**'. The choice of a variance threshold directly impacts the dimensionality of **G**', but it is interesting to note that the conservative choice of 90% typically results in **G**' being 1- to 5-dimensional, even for large families of more than 1000 structures. PCA [70] was selected for simplicity, but many other dimensionality reduction methods, both linear and non-linear (for example SciMAP [73,74]), could be substituted and would possibly further improve the dimensionality reduction step. Figure 1(c) shows the geometric feature vector encoded proteins for the 83-structure heme-dependent peroxidase family as points in the first and second principal components of **G**' which capture 94% of the original variance in **G**; the total number of principal components to reach the minimum 90% variance threshold was 2-components for the peroxidases, so **G**' was 2-dimensional in this case. Thus, PCA is able to drastically reduce the dimensionality of the geometric feature space, which is vital to the performance of most clustering algorithms.

### Step 4: identifying substructural clusters (SCs)

One approach to investigating the membership, types, and numbers of structurally related sub-groups within a larger family of proteins is to find clusters of geometrically related structures. Geometric feature vector encoding allows us to represent each protein in a family of structures as a point in feature space, and the process of finding groups or clusters of similar points in feature space can be delegated to an assortment of standard clustering methods.

To choose a clustering method, several key features were deemed important: the method should be able to identify the number of clusters, *k*, automatically; to avoid bias, no meta-data, such as species information, should be taken into account during clustering-unsupervised learning; the method should be able to identify instances where only a single cluster is sufficient to explain variation; the method should be robust to the presence of outliers; the method should be able to accommodate the presence of both very large, dense sub-groups and small, diffuse sub-groups. Methods that rely on a user-defined number of clusters, such as *k*-means, are difficult to apply to the problem of identifying significant clusters within *F*, because the number of clusters, *k*, is not known *a priori*.

To provide an automated, unbiased selection method for *k*, a Gaussian Mixture Model (GMM) approach using the MCLUST [71] package for the statistical language **R **was selected for use in this work. MCLUST incrementally adds multivariate Gaussians to the mixture model, fitting them through an iterative Expectation Maximization procedure, and assesses the Bayesian Information Criteria (BIC), while regularizing for model complexity to select a set of Gaussians that maximally explain the data, given the model complexity constraint. The GMM defines, for each data point, the probability that it belongs to the *i*th Gaussian mixture component and then a hard classification is performed to partition the data points into the mixture components from which the points were most likely to have been generated. The colors of the data points in Figure 1(c) demonstrate the hard classification, into 4 clusters, made by the GMM for the peroxidase family of proteins (EC 1.11.1.7). The final organization of clusters based upon substructural similarity shown in Figure 1(c) is the SCs output by FASST.

### Step 5: constructing consensus motifs

As a family of protein structures grows both in numbers and structural diversity, building substructural motifs for the family, as a whole becomes increasingly difficult, just as constructing HMM profiles [75] for a large set of diverse sequences is difficult. By representing each cluster identified by GMM clustering with a distinct consensus motif, the entire family can then be represented as a collection of consensus motifs which we call a motif ensemble. To build a consensus motif for a given cluster, the propagated motifs belonging to proteins within that cluster were geometrically averaged to construct an artificial consensus structure by the method used in [76]. However, if a non-artificial consensus structure is desired, picking the structure nearest the cluster centroid would also be an effective strategy for finding a representative motif for the cluster. The consensus motif construction process is repeated for each of the *k *clusters identified during **Step 4**, resulting in a motif ensemble that contains *k *consensus motifs. For example, four clusters were identified within the family of peroxidases (as shown in Figure 1(c)), and therefore the motif ensemble for the family consisted of four consensus motifs, one for each cluster.

### Step 6: estimating statistical significance

Comparing a motif to target protein structures results in a set of substructure matches of varying quality. To distinguish erroneous matches that are likely to have occurred by chance alone and therefore not functionally related to the motif from those matches which have *significant *similarity to the motif requires a statistical model of substructure similarity. The non-parametric statistical framework for matching single-substructure motifs used in previous work [42,43,72] is extended in this work to address multiple-structure motif ensembles. A detailed discussion of the single-structure statistical model can be found in [42,72] but is outlined briefly here to motivate the extension to motif ensemble statistical hypothesis testing.

#### Single-structure motif hypothesis testing

The structural uniqueness of a match of motif *S *to a target structure *T*, *M*_*S*→ *T *_can only be evaluated with respect to a background structure reference set. A reference set should be selected such that is structurally diverse and contains protein structures functionally unrelated to the motif; a detailed analysis of the choice of reference sets can be found in [42] but in this work the 95% sequence identity non-redundant PDB (nrPDB_95_) was used as a structural reference set. Given a background reference set, we can quantify whether the similarity between *M*_*S*→ *T *_and *S *is low, relative to the background, and could have occurred by chance, or that it is high, with respect to the background, and is statistically significant.

The question of whether or not a match of motif *S *to a target structure *T*, *M*_*S*→ *T *_is significantly similar to *S *can be formulated as a hypothesis test: the null hypothesis (*H*_0_) states that *S *and *T *are structurally dissimilar and that *M*_*S*→ *T *_occurred by chance; the alternative hypothesis (*H*_*A*_) states that *S *and *T *are structurally similar and *M*_*S*→ *T *_defines a sub-structural element in *T *that is analogous to *S*. Given our definition of a background structural reference set, the *p*-value of *M*_*S*→ *T*_, *p*_*S *→ *T*_, is a measure of the structurally uniqueness of *M*_*S*→ *T *_with respect to the defined background reference set. By selecting a *p*-value threshold for statistical significance, *α*, we can reject *H*_0 _for all *p*_*S *→ *T *_≤ *α *and instead accept *H*_*A *_and declare *M*_*S *→ *T *_to be statistically significant. Matching *S *versus all of the structures defined by the background reference set will yield a distribution of matches with varying levels of structural similarity to *S*, given by the LRMSD of each match to *S*. By smoothing the LRMSD distribution using the Sheather-Jones optimal bandwidth [77] we obtain a probability density function pdf(*r*) over LRMSD, *r*, for a given motif *S*; we denote this pdf as pdf(*r*; *S*).

Given pdf(*r*; *S*), the *p*-value measure of statistical significance of *M*_*S *→ *T *_can be found by calculating the probability of observing a match with LRMSD, *r*, lower than the LRMSD of *M*_*S *→ *T*_, *r*_*M*_, which can be written as *P*(*r *≤ *r*_*M*_; *S*) and defined to be: . In summary, the *p*-value of a given match of a motif to a target protein structure is calculated by comparing the match LRMSD to the population of match LRMSDs that are expected to occur by chance alone. Using this technique, matches with statistically *unusual *amounts of geometric similarity to a motif can be readily identified without making assumptions about the structure of the match distribution.

#### Motif ensemble statistical hypothesis testing

The hypothesis testing framework used for quantitating the statistical significance of matches to a standard, single-structure motif, can be extended naturally to accommodate the notion of matching an ensemble of motifs. Given a motif ensemble with *k *consensus motifs  = {*S*_1_, *S*_2_, ..., *S*_*k*_} we would like to know if the motif ensemble, , has statistically significant similarity to *T*. For each motif, *S*_*i *_∈ , we can calculate the *p*-value of matching *S*_*i *_to *T*, *ps*_*i*→ *T*_, by matching *S*_*i *_versus the background structure reference set and obtaining the probability density function over match LRMSD, *r*, for motif *S*_*i*_: pdf(*r*; *S*_*i*_). This procedure produces a *p*-value for matching each *S*_*i *_to *T*,  = {*p_S_*_1→ *T*_, *p_S_*_2→ *T*_, ..., *p_S_*_*k*→ *T*_} and, as for normal single structure motifs, an associated hypothesis test for each motif: the null hypothesis (*H*_0, *i*_)states that *S*_*i *_and *T *are structurally dissimilar and the match of *S*_*i *_to *T *occurred by chance; the alternative hypothesis (*H*_*A*, *i*_) states that *S*_*i *_and *T *are structurally similar and the match of *S*_*i *_to *T *defines a sub-structural element in *T *that is analogous to *S*_*i*_. The overall null hypothesis for a match to the motif ensemble can now be stated in terms of the individual hypothesis corresponding to each consensus motif within the motif ensemble: *H*_0 _= {*H*_0, 1_, ..., *H*_0, *k*_}.

Because the overall null hypothesis, *H*_0_, incorporates multiple hypothesis tests (*H*_0, 1_, ..., *H*_0, *k*_), each of which can introduce new false positive matches, it is crucial to use a multiple testing correction procedure to account for the presence of multiple tests and control the *family-wise error rate*. The Hochberg *p*-value correction method [78] was selected to account for the presence of multiple tests for significance; Hochberg correction is applicable when the hypothesis tests are either independent or positively correlated [79]. After Hochberg multiple testing correction has been performed on the match *p*-value, , corresponding to each hypothesis test, *H*_0, *i*_, each null hypothesis can then be independently evaluated: . If any null hypothesis, *H*_0, *i*_, is rejected, we then reject the overall null hypothesis, *H*, and consider the match between  and *T *to be statistically significant (a positive match).

## Abbreviations

FASST: Family-wise Analysis of SubStructural Templates; MESH: Motif Ensemble Statistical Hypothesis testing; SCs: Substructural Clusters; LRMSD: Least Root Mean Square Deviation; GMM: Gaussian Mixture Model; PCA: Principal Components Analysis; HMM: Hidden Markov Model; SCOP: Structural Classification Of Proteins database; EC: Enzyme Commission.

## Authors' contributions

All authors collectively conceived and designed the experiments and analyzed the resulting data. DHB, MM, and VYF contributed computational analysis tools/software. DHB performed experiments. DHB, BYC, MM, and LEK wrote the paper. All authors read and approved the final manuscript.

## Supplementary Material

Additional file 1 **Effect of many outliers on FASST for the heme-dependent peroxidases**. **(a) **FASST applied to the 83 peroxidase structures plus 50 randomly selected, functionally unrelated structures from the nrPDB_95_. Only 37 of the 50 unrelated structures contained a possible match to the motif (i.e., a substructure with compatible alternate residue labels/mutations to the motif). The peroxidase clusters maintain almost identical structure (relative to Figure 1) even though 30% of the "family" analyzed by FASST in this case consists of unrelated proteins. Unlike the peroxidase structures, the unrelated structures form sparse, normally distributed scatter with no well-defined clusters (orange points). The extreme peroxidase outlier structure [PDB:1BGP] falls at the left-most extreme of the orange cluster. **(b) **FASST applied to the heme-dependent peroxidase SCOP superfamily, including 83 structures from EC:1.11.1.7 combined with an additional 110 structures from EC:1.11.1.5 (cytochrome-c peroxidases), EC:1.11.1.6 (catalases), and EC:1.11.1.11 (L-ascorbate peroxidases). All EC:1.11.1.7 heme-dependent peroxidases reside in cluster (i) with the exception of [PDB:1BGP] which falls into the scattered cluster (ii) region; a single chloroplastic ascorbate peroxidase structure corresponding to [PDB:1IYN] also resides in cluster (i). The scattered cluster (ii) region consists almost exclusively of catalases; clusters (iii) and (iv) correspond to cytochrome-c peroxidases; cluster (v) corresponds to ascorbate peroxidases. Heme-dependent peroxidases from EC:1.11.1.7 are well-segregated from the other structurally-similar peroxidase enzymes by FASST. Click here for file

Additional file 2 **Effects of many outliers on FASST for the xylose isomerases**. **(a) **FASST applied to xylose isomerase structures plus 50 randomly selected, functionally unrelated structures from the nrPDB_95_; points are colored by automated cluster assignment. Only 30 of the 50 unrelated structures contained a possible match. All of the xylose isomerase structures form a single, dense cluster on the left side of the figure (inside the boxed region) while the 30 unrelated structures form a sparse scattered region on the right side of the figure; a single outlier xylose isomerase structure was erroneously grouped with unrelated structures (red point within the boxed region). **(b) **Magnified view of the boxed region from (a). Each point is colored identically to the phylogenetic labeling shown in (c) for comparison. **(c) **FASST applied to only xylose isomerase structures. Each structure (point) is colored according to the corresponding *Family*-level taxonomic classification. The data in (b) is simply a different projection of the same data in (c). Although the points in (b) are compressed along the y-axis (PC 2) relative to (c), the relative positions of the phylogenetic clusters is preserved. The cause of the distortion in (b) is that the optimal (maximal data variance preserving) 2-dimensional projection for both the combined set of xylose and unrelated structures differs from the optimal 2-dimensional projection for the xylose structures alone. **(d) **FASST applied to EC:5.3.1.5 (xylose isomerase) structures plus 3 additional EC:5.3.1.14 (L-rhamnose isomerases) structures which all belong to the xylose isomerase-like SCOP superfamily. Cluster (i) corresponds to all EC:5.3.1.5 structures while clusters (ii) and (iii) correspond to apo and holo structures, respectively, from EC:5.3.1.14. Click here for file

Additional file 3 **Sequence- and structure-based all-against-all analysis of the heme-dependent peroxidases**. The heme-dependent peroxidase family was combined with 50 functionally unrelated structures to illustrate the degree of intra-family similarity evident using sequence and whole structure comparison approaches. The plant and fungal enzymes both have a CCP-like fold (SCOP:48114) that differs from the mammalian enzymes. **(a) **All-against-all sequence distances using CLUSTALW for pairwise sequence alignments. Clusters labeled (i) and (ii) correspond to the plant Families *Brassicaceae *and *Fabaceae*/*Poaceae*, respectively; cluster (iii) corresponds to the unrelated nrPDB structures; cluster (iv) corresponds to the fungal Families *Psathyrellaceae*/*Tricholomataceae*; clusters (v) and (vi) correspond to the mammalian Families *Hominidae *and *Bovidae*, respectively. **(b) **All-against-all structure distances using Combinatorial Extension (CE) for whole-structure alignment. Clusters (i) and (ii) correspond to the plant and fungal structures, respectively; cluster (iii) consists of plant [PDB:1BGP] and fungal [PDB:1MNP] outliers in addition to four functionally unrelated structures; the several clusters in region (v) correspond to functionally unrelated protein; clusters in region (iv) correspond to mammalian peroxidases. **(c) **All-against-all sequence distances using CLUSTALW for pairwise alignment of all heme-dependent peroxidase SCOP superfamily structures. Cluster (i) corresponds to lactoperoxidases (EC:1.11.1.7); cluster (ii) consists of both catalases (EC:1.11.1.6) and cytochrome-c peroxidases (EC:1.11.1.5); clusters (iii) and (iv) contain plant heme-dependent peroxidases (EC:1.11.1.7); cluster (v) contains both catalases (EC:1.11.1.6) and L-ascorbate peroxidases (EC:1.11.1.11); and cluster (vi) includes only myeloperoxidases (EC:1.11.1.7). **(d) **All-against-all structure distances using CE for heme-dependent peroxidase SCOP superfamily structures. Cluster (i) corresponds to plant heme-dependent peroxidases (EC:1.11.1.7); cluster (ii) contains cytochrome-c peroxidases (EC:1.11.1.5); cluster (iii) contains L-ascorbate peroxidases (EC:1.11.1.11); cluster (iv) contains fungal heme-dependent peroxidases (EC:1.11.1.7); clusters (v) and (vi) contain catalases (EC:1.11.1.6); cluster (vii) includes catalases (EC:1.11.1.6) and cytochrome-c peroxidases (EC:1.11.1.5); and cluster (viii) consists of mammalian heme-dependent peroxidases (EC:1.11.1.7) including lactoperoxidases and myeloperoxidases. Click here for file

Additional file 4 **Sequence- and structure-based all-against-all analysis of the xylose isomerases**. The xylose isomerase family was combined with 50 functionally unrelated structures to illustrate the degree of intra-family similarity evident using sequence and whole structure comparison approaches. The xylose isomerase structures all share a common TIM-barrel fold. **(a) **All-against-all sequence distances using CLUSTALW for pairwise sequence alignments. Clusters (i), (ii), and (iii) correspond to mesophile structures from the *Streptomycetaceae*, *Micromonosporaceae*, *Micrococcaceae *Families, respectively; cluster (iv) and the 3 left-most cluster (v) points correspond to thermophile structures (Families: *Thermaceae*, *Thermotogaceae*, *Thermoanaerobacterales*, *Bacillaceae*); the remainder of cluster (v) consists of functionally unrelated structures. **(b) **All-against-all structure distances using Combinatorial Extension (CE) for whole-structure alignment. Cluster (i) is composed of the mesophile structures; the boxed region contains the thermophile structures. the remainder of cluster (ii) consists of functionally unrelated structures. **(c) **All-against-all sequence distances via CLUSTALW for xylose isomerase-like SCOP superfamily structures including EC:5.3.1.14 (L-rhamnose isomerase) and EC:5.3.1.5 (xylose isomerase) structures. Cluster (vi) corresponds to EC:5.3.1.14 structures while xylose isomerases make up the remaining clusters. **(d) **All-against-all structure distances calculated with CE for xylose isomerase-like SCOP superfamily structures. Cluster (vii) corresponds to EC:5.3.1.14 structures while xylose isomerases make up the remaining clusters. Click here for file
