# Supplementary figures and images for: Analysis of substructural variation in families of enzymatic proteins with applications to protein function prediction
Source: BMC Bioinformatics. 2010 May 11;11:242. doi: 10.1186/1471-2105-11-242 (PMC2885373; doi:10.1186/1471-2105-11-242)

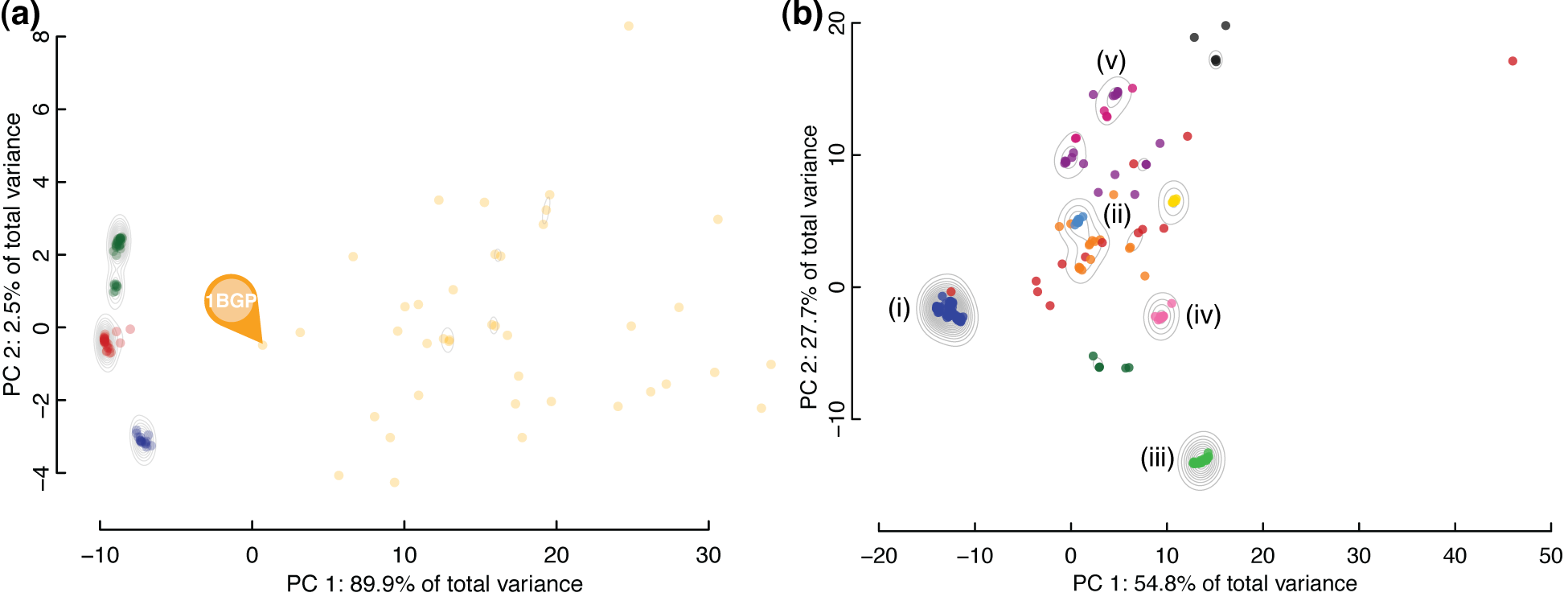

Supplement: Additional file 1 — Effect of many outliers on FASST for the heme-dependent peroxidases. (a) FASST applied to the 83 peroxidase structures plus 50 randomly selected, functionally unrelated structures from the nrPDB95. Only 37 of the 50 unrelated structures contained a possible match to the motif (i.e., a substructure with compatible alternate residue labels/mutations to the motif). The peroxidase clusters maintain almost identical structure (relative to Figure 1) even though 30% of the "family" analyzed by FASST in this case consists of unrelated proteins. Unlike the peroxidase structures, the unrelated structures form sparse, normally distributed scatter with no well-defined clusters (orange points). The extreme peroxidase outlier structure [PDB:1BGP] falls at the left-most extreme of the orange cluster. (b) FASST applied to the heme-dependent peroxidase SCOP superfamily, including 83 structures from EC:1.11.1.7 combined with an additional 110 structures from EC:1.11.1.5 (cytochrome-c peroxidases), EC:1.11.1.6 (catalases), and EC:1.11.1.11 (L-ascorbate peroxidases). All EC:1.11.1.7 heme-dependent peroxidases reside in cluster (i) with the exception of [PDB:1BGP] which falls into the scattered cluster (ii) region; a single chloroplastic ascorbate peroxidase structure corresponding to [PDB:1IYN] also resides in cluster (i). The scattered cluster (ii) region consists almost exclusively of catalases; clusters (iii) and (iv) correspond to cytochrome-c peroxidases; cluster (v) corresponds to ascorbate peroxidases. Heme-dependent peroxidases from EC:1.11.1.7 are well-segregated from the other structurally-similar peroxidase enzymes by FASST. [file 1471-2105-11-242-S1.PNG]

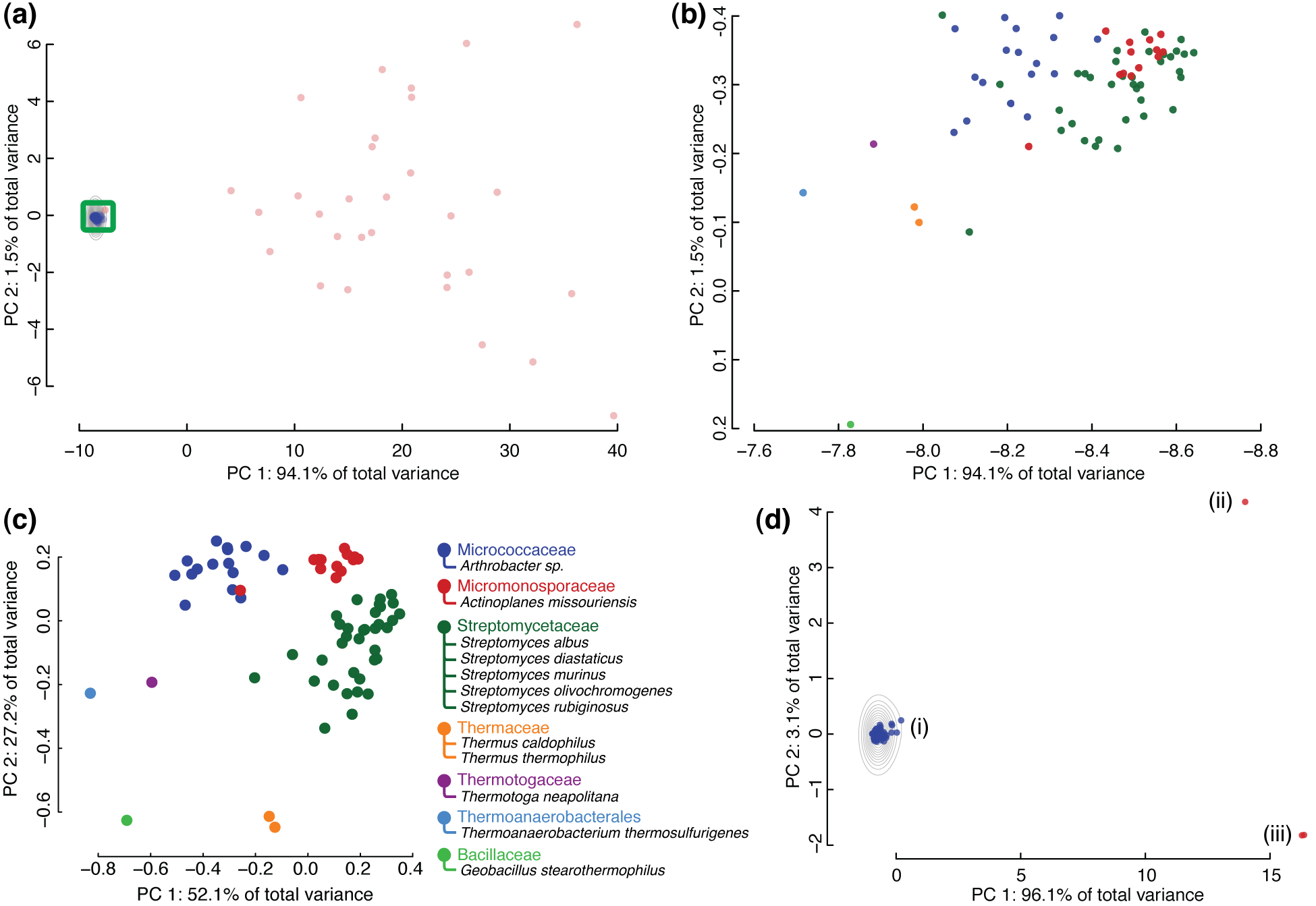

Supplement: Additional file 2 — Effects of many outliers on FASST for the xylose isomerases. (a) FASST applied to xylose isomerase structures plus 50 randomly selected, functionally unrelated structures from the nrPDB95; points are colored by automated cluster assignment. Only 30 of the 50 unrelated structures contained a possible match. All of the xylose isomerase structures form a single, dense cluster on the left side of the figure (inside the boxed region) while the 30 unrelated structures form a sparse scattered region on the right side of the figure; a single outlier xylose isomerase structure was erroneously grouped with unrelated structures (red point within the boxed region). (b) Magnified view of the boxed region from (a). Each point is colored identically to the phylogenetic labeling shown in (c) for comparison. (c) FASST applied to only xylose isomerase structures. Each structure (point) is colored according to the corresponding Family-level taxonomic classification. The data in (b) is simply a different projection of the same data in (c). Although the points in (b) are compressed along the y-axis (PC 2) relative to (c), the relative positions of the phylogenetic clusters is preserved. The cause of the distortion in (b) is that the optimal (maximal data variance preserving) 2-dimensional projection for both the combined set of xylose and unrelated structures differs from the optimal 2-dimensional projection for the xylose structures alone. (d) FASST applied to EC:5.3.1.5 (xylose isomerase) structures plus 3 additional EC:5.3.1.14 (L-rhamnose isomerases) structures which all belong to the xylose isomerase-like SCOP superfamily. Cluster (i) corresponds to all EC:5.3.1.5 structures while clusters (ii) and (iii) correspond to apo and holo structures, respectively, from EC:5.3.1.14. [file 1471-2105-11-242-S2.PNG]

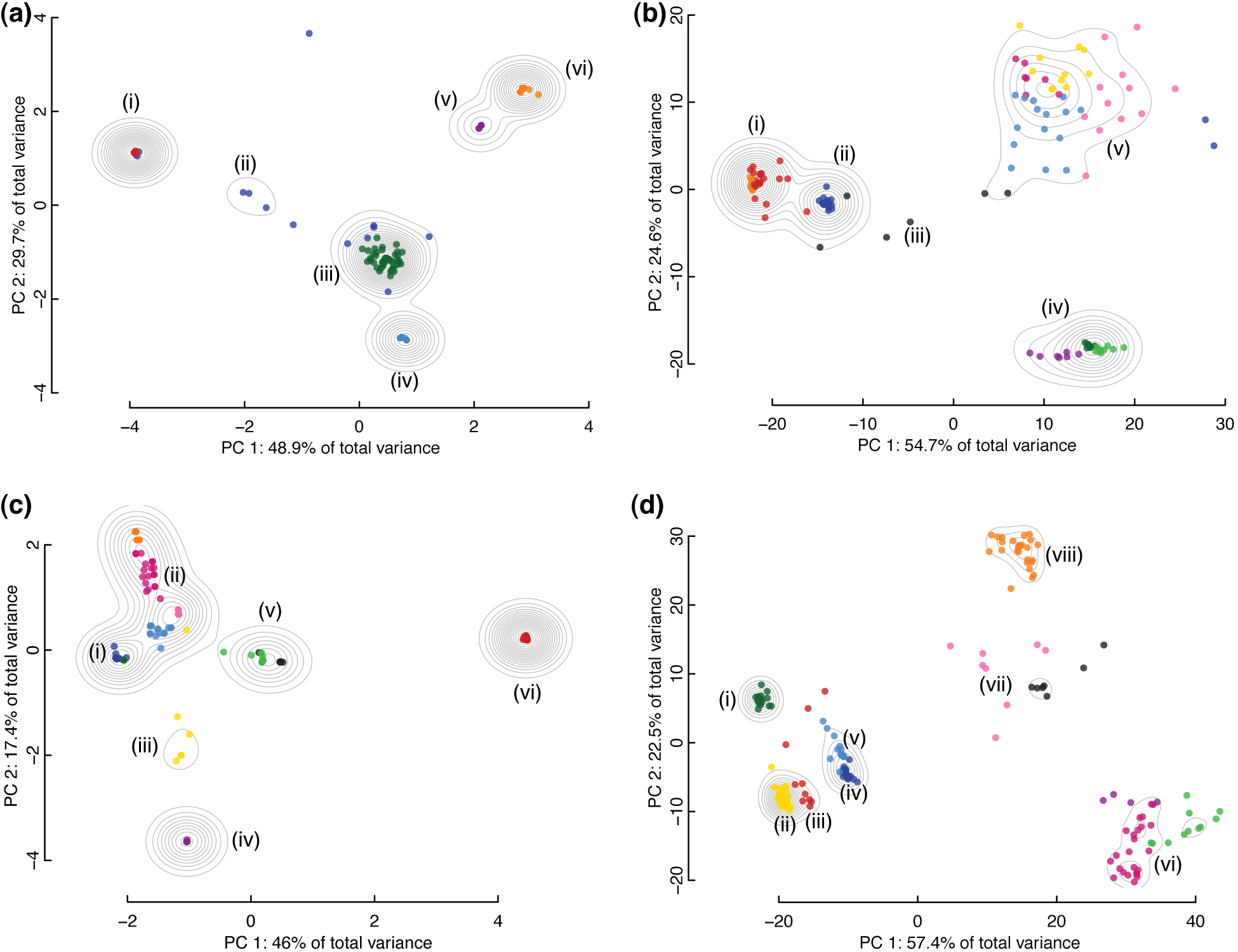

Supplement: Additional file 3 — Sequence- and structure-based all-against-all analysis of the heme-dependent peroxidases. The heme-dependent peroxidase family was combined with 50 functionally unrelated structures to illustrate the degree of intra-family similarity evident using sequence and whole structure comparison approaches. The plant and fungal enzymes both have a CCP-like fold (SCOP:48114) that differs from the mammalian enzymes. (a) All-against-all sequence distances using CLUSTALW for pairwise sequence alignments. Clusters labeled (i) and (ii) correspond to the plant Families Brassicaceae and Fabaceae/Poaceae, respectively; cluster (iii) corresponds to the unrelated nrPDB structures; cluster (iv) corresponds to the fungal Families Psathyrellaceae/Tricholomataceae; clusters (v) and (vi) correspond to the mammalian Families Hominidae and Bovidae, respectively. (b) All-against-all structure distances using Combinatorial Extension (CE) for whole-structure alignment. Clusters (i) and (ii) correspond to the plant and fungal structures, respectively; cluster (iii) consists of plant [PDB:1BGP] and fungal [PDB:1MNP] outliers in addition to four functionally unrelated structures; the several clusters in region (v) correspond to functionally unrelated protein; clusters in region (iv) correspond to mammalian peroxidases. (c) All-against-all sequence distances using CLUSTALW for pairwise alignment of all heme-dependent peroxidase SCOP superfamily structures. Cluster (i) corresponds to lactoperoxidases (EC:1.11.1.7); cluster (ii) consists of both catalases (EC:1.11.1.6) and cytochrome-c peroxidases (EC:1.11.1.5); clusters (iii) and (iv) contain plant heme-dependent peroxidases (EC:1.11.1.7); cluster (v) contains both catalases (EC:1.11.1.6) and L-ascorbate peroxidases (EC:1.11.1.11); and cluster (vi) includes only myeloperoxidases (EC:1.11.1.7). (d) All-against-all structure distances using CE for heme-dependent peroxidase SCOP superfamily structures. Cluster (i) corresponds to plant heme-dependent per [file 1471-2105-11-242-S3.PNG]

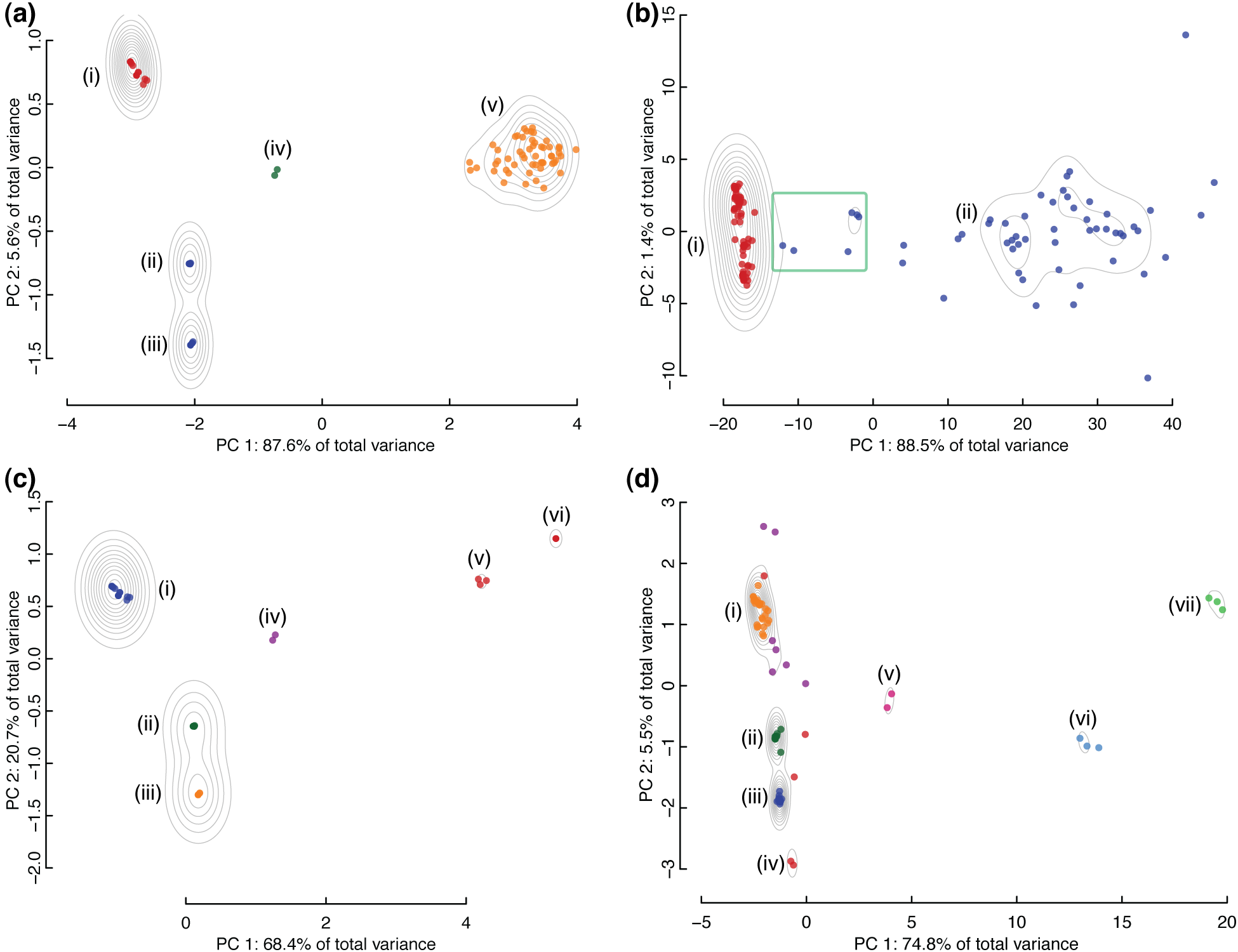

Supplement: Additional file 4 — Sequence- and structure-based all-against-all analysis of the xylose isomerases. The xylose isomerase family was combined with 50 functionally unrelated structures to illustrate the degree of intra-family similarity evident using sequence and whole structure comparison approaches. The xylose isomerase structures all share a common TIM-barrel fold. (a) All-against-all sequence distances using CLUSTALW for pairwise sequence alignments. Clusters (i), (ii), and (iii) correspond to mesophile structures from the Streptomycetaceae, Micromonosporaceae, Micrococcaceae Families, respectively; cluster (iv) and the 3 left-most cluster (v) points correspond to thermophile structures (Families: Thermaceae, Thermotogaceae, Thermoanaerobacterales, Bacillaceae); the remainder of cluster (v) consists of functionally unrelated structures. (b) All-against-all structure distances using Combinatorial Extension (CE) for whole-structure alignment. Cluster (i) is composed of the mesophile structures; the boxed region contains the thermophile structures. the remainder of cluster (ii) consists of functionally unrelated structures. (c) All-against-all sequence distances via CLUSTALW for xylose isomerase-like SCOP superfamily structures including EC:5.3.1.14 (L-rhamnose isomerase) and EC:5.3.1.5 (xylose isomerase) structures. Cluster (vi) corresponds to EC:5.3.1.14 structures while xylose isomerases make up the remaining clusters. (d) All-against-all structure distances calculated with CE for xylose isomerase-like SCOP superfamily structures. Cluster (vii) corresponds to EC:5.3.1.14 structures while xylose isomerases make up the remaining clusters. [file 1471-2105-11-242-S4.PNG]
